# Genomic evidence that microbial carbon degradation is dominated by iron redox metabolism in thawing permafrost

**DOI:** 10.1038/s43705-023-00326-5

**Published:** 2023-11-23

**Authors:** Karl J. Romanowicz, Byron C. Crump, George W. Kling

**Affiliations:** 1 Department of Ecology and Evolutionary Biology, University of Michigan, Ann Arbor, MI USA; 2 College of Earth, Ocean, and Atmospheric Sciences, Oregon State University, Corvallis, OR USA

**Keywords:** Microbial ecology, Soil microbiology, Climate-change impacts

## Abstract

Microorganisms drive many aspects of organic carbon cycling in thawing permafrost soils, but the compositional trajectory of the post-thaw microbiome and its metabolic activity remain uncertain, which limits our ability to predict permafrost–climate feedbacks in a warming world. Using quantitative metabarcoding and metagenomic sequencing, we determined relative and absolute changes in microbiome composition and functional gene abundance during thaw incubations of wet sedge tundra collected from northern Alaska, USA. Organic soils from the tundra active-layer (0–50 cm), transition-zone (50–70 cm), and permafrost (70+ cm) depths were incubated under reducing conditions at 4 °C for 30 days to mimic an extended thaw duration. Following extended thaw, we found that iron (Fe)-cycling Gammaproteobacteria, specifically the heterotrophic Fe(III)-reducing *Rhodoferax* sp. and chemoautotrophic Fe(II)-oxidizing *Gallionella* sp., increased by 3–5 orders of magnitude in absolute abundance within the transition-zone and permafrost microbiomes, accounting for 65% of community abundance. We also found that the abundance of genes for Fe(III) reduction (e.g., MtrE) and Fe(II) oxidation (e.g., Cyc1) increased concurrently with genes for benzoate degradation and pyruvate metabolism, in which pyruvate is used to generate acetate that can be oxidized, along with benzoate, to CO_2_ when coupled with Fe(III) reduction. Gene abundance for CH_4_ metabolism decreased following extended thaw, suggesting dissimilatory Fe(III) reduction suppresses acetoclastic methanogenesis under reducing conditions. Our genomic evidence indicates that microbial carbon degradation is dominated by iron redox metabolism via an increase in gene abundance associated with Fe(III) reduction and Fe(II) oxidation during initial permafrost thaw, likely increasing microbial respiration while suppressing methanogenesis in wet sedge tundra.

## Introduction

Permafrost soils contain an estimated 1600 Pg of organic carbon (OC), accounting for nearly 60% of the global belowground OC pool, the majority of which is stored in a perennially frozen state [1, 2]. However, recent warming in the Arctic has increased soil temperatures and thawed large regions of permafrost [3, 4]. Warmer soil temperatures, along with earlier spring thaw and later fall freeze-up, have also contributed to an increase in annual thaw depth and an extended duration of annual thaw along tundra soil profiles [5–7]. These warming-induced changes to permafrost will likely increase microbial activity following thaw, leading to more oxidation of previously frozen OC and the release of carbon dioxide (CO_2_) and methane (CH_4_) to the atmosphere [8–10].

The fate of the OC pool in permafrost soils will largely be determined by changes in the soil microbiome and its activity following permafrost thaw [11–14], yet major uncertainties remain in our current understanding of the microbial response to permafrost thaw. These uncertainties include both short-term and long-term impacts of soil warming on the composition of the permafrost microbiome and its metabolic response to thaw. Previous studies that document the response of permafrost microbiomes to short-term warming (i.e., days) in laboratory-based incubations have shown rapid, substantial shifts in composition, gene abundance, and gene expression that were used to predict post-thaw biogeochemical rates of CO_2_ and CH_4_ production [15–18]. Conversely, results from long-term soil warming experiments in tundra ecosystems show little or no change in the composition of the permafrost microbiomes following years of passive surface warming that extends thaw depth and duration into the permafrost [19–21]. The rapid response of the permafrost microbiome to short-term laboratory warming may be due to changes in the quantity and quality of substrates available for microbial metabolism released from newly-thawed permafrost [22]. In contrast, long-term warming experiments extending thaw into the permafrost likely prevent substantial compositional changes in the post-thaw community through persistent substrate depletion during years of passive warming [23]. However, a recent field study revealed that the composition of the permafrost microbiome is strongly correlated with the duration of annual thaw along the soil depth profile [7], suggesting that different outcomes between short- and long-term warming experiments may be due, in part, to different durations over which the permafrost soils are exposed to thaw.

The microbial response to permafrost thaw is also influenced by soil redox states [24–26], where the majority of OC degradation in thawing permafrost soils occurs under reducing conditions that promote anaerobic and fermentative microbial processes [27–31]. Anaerobic respiration in thawing soils is tightly coupled to the cycling of iron (Fe), which can serve as a terminal electron acceptor when coupled with the oxidation of OC, resulting in CO_2_ formation [32, 33]. Tundra soils are often rich in reactive Fe(III) minerals and dissolved Fe(III) that promote the oxidation of OC to CO_2_ under reducing conditions, accounting for an estimated 40 to 60% of total ecosystem respiration in low-lying, water-saturated tundra regions [34]. Fe-mediated microbial growth can also suppress CH_4_ production when Fe(III)-reducing bacteria outcompete methanogenic archaea for substrates [24, 35, 36]. Thus, it is necessary to identify how the pathways of OC degradation will be affected by Fe-mediated processes during short-term and extended thaw durations to better understand how future thaw may contribute to CO_2_ and CH_4_ production and emissions from widespread Fe-rich tundra soils.

Here, we simulated thaw along the depth profile of an Fe-rich organic tundra soil using frozen soil samples from the active-layer, transition-zone, and permafrost depths that were gradually thawed to 4 °C and incubated under reducing conditions for an extended duration of 30 days. These incubation conditions reflect a representative thaw temperature and soil redox state at depth in a newly-thawed soil, and maintained for longer than the minimum duration of annual thaw in the transition zone (~22 days) as measured from long-term thaw depth surveys at our field site [7]. We determined the extent of initial differences in microbiome composition between each soil layer along the soil profile and identified the dominant microbial taxa and their metabolic pathways of OC degradation during short-term (7-day) and extended (30-day) thaw duration by measuring relative and absolute changes in microbiome composition and functional gene abundance during the incubation experiment.

## Methods

### Study site and soil core collection

Soil core samples were collected on 19 July 2019 in wet sedge tundra at Imnavait Creek (68°36'35.36“N 149°18'29.80“W), located along the trans-Alaskan pipeline on the North Slope of the Brooks Range in northern Alaska, USA. A detailed description of the study site is given elsewhere [7, 31]. Within the wet sedge tundra, a soil core (10 cm diameter × 1 m length) was collected using a SIPRE corer with carbide bits from a location representative of the valley bottom (i.e., roughly equidistant between the bottom of the hillslope and the stream). The soil core was scraped horizontally, perpendicular to the depth axis, using aseptic techniques in the field to remove outer layers of soil and then separated into 30-cm increments, wrapped in aluminum foil, labeled by depth to reconstruct the soil profile in the lab, placed in a cooler with ice, and immediately frozen at −80 °C upon return to Toolik Field Station.

### Soil incubation design

Soil samples (~50 g, wet mass) were taken from three distinct soil layers representing the active layer (AL, 0–50 cm), transition zone (TZ, 50–70 cm), and permafrost (PF, 70+ cm) following homogenization of each (frozen) soil layer and placed in 250 mL amber jars in triplicate (*N* = 9). Each incubation jar also contained 50 ml of deionized (sterile) water and the soil samples were mixed with the deionized water to create soil slurries that were then incubated at 4 °C in the dark for the duration of the experiment. The soil layer depths used (AL, TZ, PF) were determined from long-term thaw depth surveys described elsewhere [7, 37]. Soil subsamples (~2 g, wet mass) were collected frozen from each soil layer replicate prior to starting the incubation experiment to represent time point zero (T0). Incubation jars were then sealed with an airtight lid containing a septum and purged with N_2_ to simulate reducing conditions in the soils. Additional soil subsamples (~2 g, wet mass) were collected at 7 days (T7) and 30 days (T30) following thaw and immediately frozen at −80 °C until DNA extraction.

### DNA extraction and sequencing

Microbial DNA was extracted from soil subsamples collected at each incubation time point (*N* = 27) using the RNeasy PowerSoil DNA Elution Accessory kit (Qiagen, Hilden, Germany). 16S rRNA genes were PCR-amplified from genomic DNA using dual-barcoded primers 515f-806r [38, 39]. PCR amplicons (*N* = 27) were pooled into a single library and submitted to the University of Michigan Microbiome Core for high-throughput sequencing on the Illumina MiSeq platform. Metagenome reads were generated from genomic DNA libraries (Nextera XT; *N* = 27) and all libraries were sequenced at the University of Michigan Advanced Genomics Core on the Illumina NextSeq 2000 platform using high-output 150 cycle paired-end reads.

### 16S rRNA gene analysis

Sequencing data from 16S amplicons were analyzed using QIIME2 (v. 2020.11) [40] on the University of Michigan Great Lakes High Performance Computing Cluster. Raw forward and reverse sequencing reads were quality filtered with DADA2 [41]. Taxonomy was assigned to amplicon sequence variants (ASVs) using scikit-learn naïve Bayes taxonomy classifier [42] against the SILVA sequence database (v. 138) [43]. See also the 16S rRNA sequencing reads summary (Table S1).

### Metagenome analysis

DNA sequences were trimmed and quality-filtered with the BBDuk algorithm from BBMap [44] with dereplication. Quality-controlled metagenome reads from all sample were co-assembled using MEGAHIT [45], indexed using Bowtie2 [46], with short reads from each sample individually mapped to the indexed contigs using BBMap [44]. A contigs database was created from the co-assembly using Anvi’o [47] with the abundance information for all contigs merged into an Anvi’o profile. Coding sequences (CDS) within the contigs database were predicted with PRODIGAL [48] while HMMER [49] was used to search for and tabulate the occurrence of single-copy housekeeping genes for bacteria and archaea from two collections [50, 51]. CDS were annotated to the Kyoto Encyclopedia of Genes and Genomes database (KEGG) [52] using the online GhostKOALA program [53] and imported into the contigs database. CDS were also annotated using FeGenie [54], which provides a comprehensive database of HMMs based on genes associated with Fe metabolism, Fe acquisition, and Fe cycling in bacteria and archaea. Counts per annotation were normalized to genes per million (GPM) [55] to reduce biases associated with library size and CDS length. See also the metagenome sequencing reads summary (Table S2).

### Internal standard for absolute gene abundance

The addition of DNA from the internal standard *Thermus thermophilus*, a bacterial taxon unlikely to be found in soil, prior to the DNA extraction step allows for the quantitative comparison of the absolute abundance of 16S rRNA gene copies (taxonomic abundance) and KEGG ortholog (KO) gene copies (functional abundance) across samples. Here, we followed methods described in [56–58] to spike 5.5 ng of *T. thermophilus* HB-8 DNA (purchased from ATCC; www.atcc.org; #27634D-5) into our soil samples prior to extraction. From this DNA spike-in, we calculated the absolute abundance of 16S rRNA and KO gene copies per gram of soil recovered from each sample relative to the number of recovered gene copies associated with the internal standard in the sequencing datasets. See the Supplemental Materials for additional methods. Previous studies have demonstrated that adding a DNA internal standard to soil prior to DNA extraction is an effective quantitative method for comparing bacterial taxonomic and functional gene abundances across samples [56, 57, 59].

### Statistical analysis

All statistical analyses were conducted in R (v. 4.1.2) [60] and considered significant at *p* < 0.05. Multivariate statistical analysis of the 16S amplicon sequencing data was conducted using the R “microeco” package (v. 0.12) [61] and the “vegan” package (v. 2.6–2) [62]. The Shannon diversity index (H’) was calculated using total read counts per sample (unrarefied; Table S1) and assessed via two-way ANOVA with post hoc Tukey honestly significant different (HSD) tests to determine the effects of soil layer and incubation time point on taxonomic alpha diversity. Total reads per sample were then rarefied to the lowest sample read count (71,245 reads; Table S1). ASV abundance was calculated from the rarefied read counts per sample using Bray-Curtis dissimilarity and analyzed via permutational multivariate analysis of variance (PERMANOVA) to determine the effects of soil layer, incubation time, and their interactions on the composition of each soil layer microbiome during the incubation experiment. Differences in ASV abundance by soil layers between all incubation time points were visualized through unconstrained principal coordinate analysis (PCoA) ordination using the “ggplot2” package (v. 3.3.6) [63]. Pairwise comparisons among dominant microbial phyla within each soil layer were conducted between each incubation time point via two-way ANOVA with post hoc Tukey HSD tests to determine if the relative abundance of each phylum changed following thaw in the incubation experiment.

Functional gene abundance within each soil-layer microbiome at each incubation time point was determined from the GPM-normalized metagenomes based on the relative abundance of genes annotated to KEGG tiers II-IV [52] or to a comprehensive database of HMMs incorporating protein families associated with Fe metabolism, Fe acquisition, and Fe cycling in FeGenie [54]. Pairwise comparisons among functional genes within each soil layer microbiome were conducted between each incubation time point relative to T0 via two-way ANOVA with post hoc Tukey HSD tests to determine if the relative abundance of functional genes changed following thaw. The Shannon diversity index (H’) was calculated (“vegan” package) using total mapped reads per sample (Table S2) and assessed via two-way ANOVA with post hoc Tukey HSD tests to determine the effect of soil layer on functional gene alpha diversity. Beta diversity treatment effects were estimated using PERMANOVA based on Bray-Curtis dissimilarity distance between GPM-normalized functional gene counts. Pairwise comparisons for Fe-associated and OC degrading microbial pathways were conducted via two-way ANOVA with post hoc Tukey HSD tests to determine if the relative and absolute abundance of Fe-associated or OC degrading genes changed following thaw in the incubation experiment.

## Results

### Taxonomic diversity

Soil microbiome analysis of 16S rRNA gene amplicons resolved into amplicon sequence variants (ASVs) showed statistical differences in taxonomic alpha diversity between distinct soil layers along the profile of our soil core and between incubation time points within each soil layer using Shannon (H’) diversity metrics (Fig. S1). Specifically, there was significantly greater taxonomic alpha diversity (H’) in the active-layer microbiome compared to the transition-zone and permafrost microbiomes at each incubation time point, with no difference between the transition-zone and permafrost microbiomes at each time point (Fig. S1). Within each soil layer microbiome, there was no difference in taxonomic H’ between the initial (T0) and short-term (T7) thaw duration (Fig. S1). However, there was a significant decrease in taxonomic H’ following extended thaw duration (T30) compared to T0 within all three soil layer microbiomes (−15.4% AL, −66.5% TZ, −64.6% PF; Fig. S1).

**Fig. 1:**
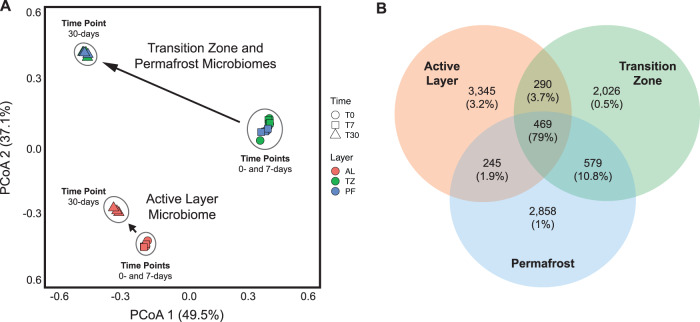
Changes in taxonomic diversity by soil layer during extended thaw incubation. Beta diversity (**A**) ordination from principal coordinate analysis (PCoA) based on Bray-Curtis dissimilarity in the relative abundance of 16S rRNA amplicon sequence variants (ASVs) within each soil layer microbiome. Each point represents an individual sampling replicate from each soil layer collected at each incubation time point during the experiment (*N* = 27). **B** Venn diagram of unique and shared 16S ASVs for each soil layer microbiome. The total ASVs of each soil layer and those ASVs shared between soil layers are shown as counts. The relative abundance of ASVs associated with each count value is shown as a percentage in parentheses.

Beta diversity differences in ASV abundance between soil layers and between incubation time points were visualized through PCoA ordination based on Bray-Curtis dissimilarity (Fig. 1A). There were four notable trends from the PCoA ordination. First, the active-layer microbiome was significantly distinct from the transition-zone and permafrost microbiomes throughout the incubation experiment, while the transition-zone and permafrost microbiomes were indistinguishable from each other based on their shared abundance of ASVs (Fig. 1A). Second, there were no significant differences in the beta diversity of ASV abundance between incubation time points T0 and T7 in any soil layer, indicating that there was no significant change following the 7-day short-term thaw duration (Fig. 1A). Third, the active-layer microbiome significantly shifted in composition at time point T30 compared to T0 or T7 (Fig. 1A). Fourth, the transition-zone and permafrost microbiomes both shifted significantly in composition by T30, but the magnitude of these shifts was much greater than observed in the active-layer microbiome, indicating these microbiomes deeper in the soil were more susceptible to whole-community compositional shifts following extended thaw (Fig. 1A).

The Venn diagram indicates that 469 ASVs were shared between all three soil layers, which represents 79% of all ASV relative abundance (Fig. 1B). An additional 1114 ASVs were shared between at least two soil layers, with more ASVs shared between the transition-zone and permafrost microbiomes (579 ASVs) than between the active layer and transition-zone (290 ASVs) or active layer and permafrost (245 ASVs) microbiomes (Fig. 1B). Notably, these 1583 ASVs shared between two or more soil layers account for 95.5% of all ASV relative abundance shared between soil layers but there were still an additional 8229 ASVs unique to only one soil layer. The active-layer microbiome had the greatest abundance of unique ASVs (3345) compared to the transition-zone microbiome (2026) and permafrost microbiome (2858) that together accounted for the remaining 4.7% of all ASV relative abundance (Fig. 1B).

### Taxonomic abundance

**Fig. 2:**
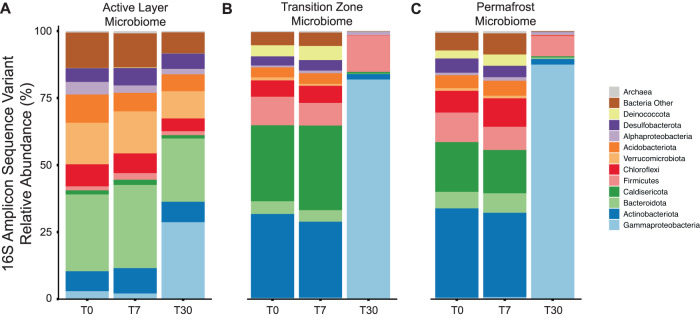
Changes in the relative abundance of microbial taxa by soil layer during extended thaw incubation. Relative abundance (mean %) of dominant bacterial phyla and archaea within the (**A**) active-layer microbiome, (**B**) transition-zone microbiome, and (**C**) permafrost microbiome derived from the 16S rRNA amplicon sequence variants (ASVs). Pseudomonadota (formerly Proteobacteria) are resolved to the class level.

Using taxonomic annotations of the 16S rRNA ASVs for the experimental thaw over time, we found a significant increase in the relative abundance of Gammaproteobacteria in the active layer (+25.8%), transition-zone (+81.5%), and permafrost (+87.0%) microbiomes after 30 days of thaw relative to initial composition at T0 (Fig. 2). Within the active-layer microbiome, there also was a significant decrease in the relative abundance of Verrucomicrobiota (−5.4%) and Chloroflexi (−3.4%) at T30 relative to T0 (Fig. 2A). Within the transition-zone and permafrost microbiomes, there was a significant decrease in nearly all other phyla at T30 relative to T0 (Fig. 2B, C and Table S3).

Using sequence counts recovered from the internal standard *Thermus thermophilus* (Fig. S2A), the calculated absolute abundance of non-*T. thermophilus* 16S rRNA gene copies (g soil)^−1^ increased more than two-orders of magnitude by T30 compared to T0 within the transition-zone and permafrost microbiomes (Fig. S2B). In contrast, the active-layer microbiome only experienced proportional shifts in the absolute abundance of 16S rRNA gene copies associated with dominant taxa rather than a significant increase in overall absolute abundance of 16S rRNA gene copies between incubation time points (Fig. S2B). Within the transition-zone and permafrost microbiomes, there was a significant increase in the absolute abundance of Firmicutes (Log_10_ fold-change = +1.5 TZ and PF), Alphaproteobacteria (+1.6 TZ and PF), and Gammaproteobacteria (+3.7 TZ & PF) by T30 compared to T0 (Table S4). There was no significant change in absolute gene copy abundance between incubation time points T0 and T7 within any soil layer microbiome (Table S4).

The substantial increase in the relative abundance of Gammaproteobacteria within all soil layers by T30 was mostly attributed to the growth of *Rhodoferax* sp. (+10.3% AL, +62.3% TZ, +62.6% PF; Table 1). The cultivated lineage most closely related to the abundant ASV annotated as *Rhodoferax* sp. was the Fe(III)-reducing bacterium *R. ferrireducens* (BLASTN: percent identity = 100%; e-value = 2e^−127^). *Gallionella* sp., a known Fe(II)-oxidizing bacterium, also contributed to the significant increase in Gammaproteobacteria abundance in all soil layers (+7.7% AL, +1.6% TZ, +3.1% PF; Table 1). The Fe(III)-reducing *Geobacter* sp. (Desulfobacterota) remained at low relative abundance throughout the incubation experiment in all soil layers (<0.8% AL, <0.1% TZ, <0.1% PF; Table 1). Overall, the increase of Fe-cycling taxa accounted for 20% of the total relative abundance of the active-layer microbiome and 65% of the transition-zone and permafrost microbiomes by the end of the incubation (Table 1).

**Table 1:** Relative abundance (mean % ± SD) of Fe(III)-reducing and Fe(II)-oxidizing bacterial taxa recovered from 16S rRNA amplicon sequence variants (ASVs).

Soil layer and timepoint	Fe(III)-reduction				Fe(II)-oxidation	
	*Rhodoferax* sp.		*Geobacter* sp.		*Gallionella* sp.	
	Relative abundance (%)	% Change from T0	Relative abundance (%)	% Change from T0	Relative abundance (%)	% Change from T0
Active Layer						
T0	0.1 ± 0.0	–	0.2 ± 0.1	–	0.3 ± 0.2	–
T7	0.5 ± 0.3	0.4	0.3 ± 0.1	0.1	0.3 ± 0.2	0.0
T30	**10.4** ± **0.8**	**10.3**	0.8 ± 0.6	0.6	**8.0** ± **5.2**	**7.7**
Transition zone						
T0	<0.1	–	<0.1	–	<0.1	–
T7	<0.1	0.0	<0.1	0.0	<0.1	0.0
T30	**62.3** ± **13.7**	**62.3**	<0.1	0.0	**1.6** ± **0.8**	**1.6**
Permafrost						
T0	<0.1	–	<0.1	–	<0.1	–
T7	<0.1	0.0	<0.1	0.0	<0.1	0.0
T30	**62.6** ± **9.1**	**62.6**	<0.1	0.0	**3.1** ± **1.7**	**3.1**

Percent change values denote the mean change in relative abundance (%) for each Fe-related taxon compared to incubation time point T0 within each soil layer microbiome. Bold values indicate a significant difference in mean relative abundance for each Fe-related taxon compared to incubation time point T0 within each soil layer microbiome (ANOVA; *p* < 0.05).

**Fig. 3:**
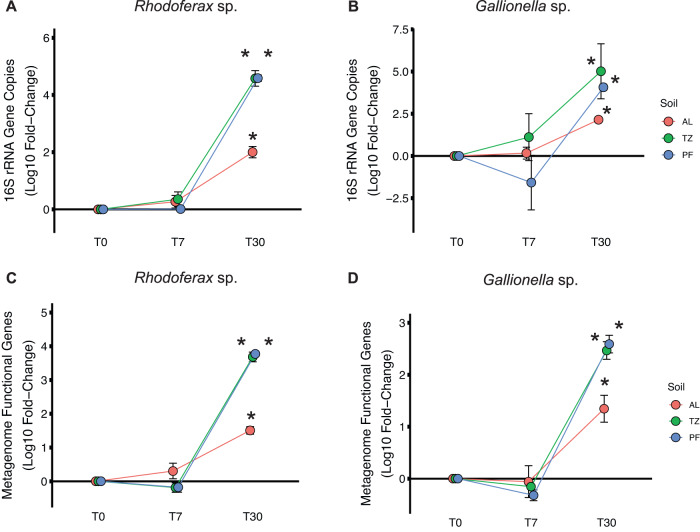
Changes in absolute abundance of gene copies associated with Fe-cycling taxa by soil layer during extended thaw incubation. Log_10_ fold-change (mean ± SE) in absolute abundance of (**A**, **B**) 16S rRNA gene copies and metagenome functional genes (**C**, **D**) for the dominant Fe(III)-reducing bacterial taxon (**A**, **C**) *Rhodoferax* sp. and dominant Fe(II)-oxidizing bacterial taxon (**B**, **D**) *Gallionella* sp. compared to incubation sampling time point T0 within each soil layer microbiome. Absolute abundance values were derived from 16S rRNA amplicon sequencing and metagenome sequencing, respectively, relative to *Thermus thermophilus* internal standard recovered from each sequencing effort. An asterisk indicates a significant Log_10_ fold-change compared to incubation time point T0 within each soil layer microbiome (ANOVA; *p* < 0.05).

The absolute abundance of 16S rRNA gene copies associated with Fe-cycling taxa also increased by several orders of magnitude following extended thaw duration (T30) compared to initial values at T0 within each soil layer microbiome (Table S5). Specifically, there was a significant increase in the absolute abundance of 16S rRNA gene copies associated with *Rhodoferax* sp. in the active-layer (Log_10_ fold-change = +2.1), transition-zone (+4.4), and permafrost (+4.6) microbiomes by T30 compared to T0 (Fig. 3A). The absolute abundance of 16S rRNA gene copies associated with the Fe(II)-oxidizing *Gallionella* sp. also increased significantly after 30 days of thaw in the active layer (Log_10_ fold-change = +2.2), transition-zone (+3.6), and permafrost (+4.1) microbiomes (Fig. 3B). Together, these changes represent an increase in the absolute abundance of 16S rRNA gene copies associated with *Rhodoferax* sp. (Fig. 3A) and *Gallionella* sp. (Fig. 3B) of more than two orders of magnitude in the active layer microbiome and up to four orders of magnitude in the transition-zone and permafrost microbiomes after 30 days of thaw. However, like the relative abundance results, the absolute abundance of 16S rRNA gene copies associated with the Fe(III)-reducing *Geobacter* sp. remained unchanged by T30 in all soil layers (Table S5).

### Functional gene diversity

Metagenome analysis revealed that functional gene diversity in the active-layer microbiome was significantly greater than in the transition-zone or permafrost microbiomes at all incubation time points (Shannon H’; Fig. S3). However, within the active-layer microbiome, there was no significant change in functional gene H’ during short-term (T7) or extended (T30) thaw duration compared to T0 (Fig. S3). In contrast, the functional gene H’ of the transition-zone and permafrost microbiomes significantly decreased from T0 to T30 (−8.7% TZ, −6.9% PF; Fig. S3), concurrent with the observed decline in taxonomic diversity within these same microbiomes (Fig. S1).

### Functional gene abundance

Metagenome analysis revealed that the relative abundance of KEGG-annotated functional genes was statistically similar between incubation time points T0 and T7 but differed significantly from time point T30 within each soil layer microbiome (Fig. S4). At the KEGG tier III level (sub-categories within tier II), half of the functional gene categories in the active-layer microbiome (11 of 22) and most of the functional gene categories in the transition-zone microbiome (20 of 22) and permafrost microbiome (15 of 22) differed significantly after 30 days compared to initial values at T0 (Fig. S4). The relative abundance of KEGG orthologs (KOs) annotated to *Rhodoferax* sp. represented only 0.1–0.3% of total KOs across all soil layers at T0 but increased significantly to 9.3% in the active-layer microbiome, 49.5% in the transition-zone microbiome, and 48.4% of total KOs in the permafrost microbiome by T30. This represents an increase of +1.2 to +4.0 (Log_10_ fold-change) in absolute abundance of functional genes associated with *Rhodoferax* sp. per gram of soil extracted (Fig. 3C). Likewise, the relative abundance of KOs annotated to *Gallionella* sp. increased significantly from <0.1% of total KOs in the metagenomes at T0 to 2.5% in the active-layer microbiome, 1.1% in the transition-zone microbiome, and 1.6% of total KOs in the permafrost microbiome by T30. This represents an increase of +1.4 to +2.9 (Log_10_ fold-change) in the absolute abundance of functional genes associated with *Gallionella* sp. per gram of soil extracted (Fig. 3D). Again, the absolute abundance of functional genes associated with *Geobacter* sp. remained unchanged by T30 in all soil layers (Table S5).

### Fe-associated gene abundance

**Fig. 4:**
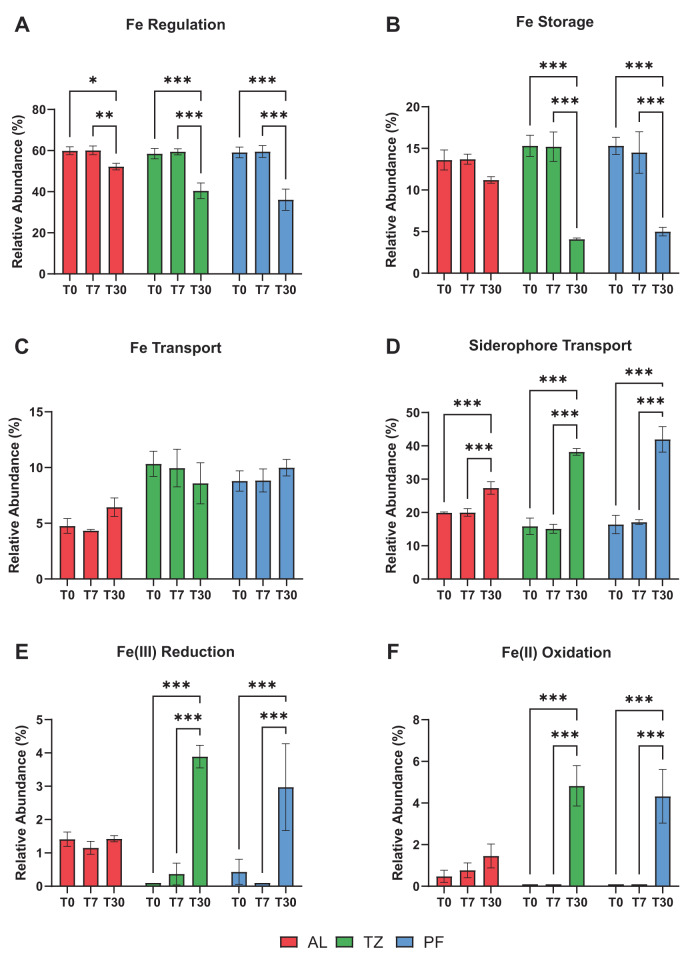
Changes in Fe-associated gene abundance by soil layer during extended thaw incubation. Relative abundance (mean % ± SD) of iron (Fe)-related gene pathways including (**A**) Fe regulation, (**B**) Fe storage, (**C**) Fe transport, (**D**) siderophore transport, (**E**) Fe(III) reduction, and (**F**) Fe(II) oxidation derived from the active layer, transition zone, and permafrost metagenomes. Significant differences were determined between incubation time points within each soil layer microbiome (ANOVA; ***p < 0.001, **p < 0.01, *p < 0.05).

Genes associated with Fe metabolism (Fe regulation, Fe storage), Fe acquisition (Fe transport, Fe acquisition via siderophore production), and Fe cycling (Fe(III) reduction, Fe(II) oxidation) were derived from protein families incorporated in hidden Markov models (HMMs) in FeGenie [54]. See Table S7 for a summary of the number of unique protein families incorporated in the HMMs for each FeGenie category. Here, the relative abundance of genes associated with Fe regulation (Fig. 4A) and Fe storage (Fig. 4B) decreased significantly from T0 to T30 in all soil layer metagenomes, except for Fe storage genes in the active layer metagenome (Fig. 4B). The relative abundance of Fe transport genes did not change between sampling points in any soil layer metagenome (Fig. 4C). However, the relative abundance of genes associated with Fe acquisition via siderophore production increased significantly by T30 in all soil layer metagenomes (+7.5% AL, +22.4% TZ, +25.5% PF; Fig. 4D). The relative abundance of genes associated with Fe(III) reduction also increased significantly by T30, but only in the transition-zone and permafrost metagenomes (+3.9% TZ, +2.6% PF; Fig. 4E). Likewise, the relative abundance of genes associated with Fe(II) oxidation increased significantly by T30 in the transition-zone and permafrost metagenomes (+4.8% TZ, +4.3% PF; Fig. 4F).

### Carbon degradation gene abundance

**Fig. 5:**
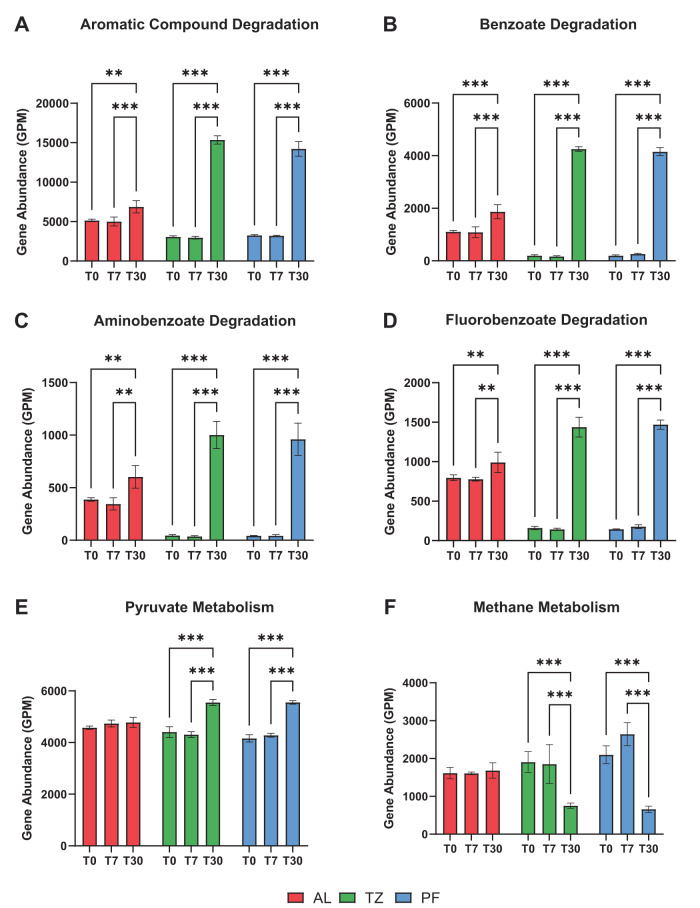
Changes in carbon degradation gene abundance by soil layer during extended thaw incubation. Normalized gene abundance (GPM; mean ± SD) of KEGG Tier IV metabolism pathways related to organic carbon degradation including (**A**) aromatic compound degradation, (**B**) benzoate degradation, (**C**) aminobenzoate degradation, (**D**) fluorobenzoate degradation, (**E**) pyruvate metabolism, and (**F**) methane metabolism derived from the active-layer, transition-zone, and permafrost metagenomes. Significant differences were determined between incubation time points within each soil layer microbiome (ANOVA; ***p < 0.001, **p < 0.01, *p < 0.05).

Genes associated with the degradation of aromatic compounds (197 genes), benzoate degradation (28 genes), aminobenzoate degradation (37 genes), fluorobenzoate degradation (6 genes), pyruvate metabolism (31 genes), and methane metabolism (45 genes) were derived from KEGG annotations at the tier IV level. See Table S8 for a summary of the number of unique genes included in each KO category associated with carbon degradation. Here, genes associated with the degradation of aromatic compounds increased significantly by T30 relative to T0 in all soil layers (Fig. 5A), representing an increase of +0.3 (AL), +1.6 (TZ), and +1.4 (PF) in absolute abundance of gene copies per gram of soil (Log_10_ fold-change; Table S6). Similarly, genes associated with benzoate degradation, aminobenzoate degradation, and fluorobenzoate degradation increased significantly by T30 relative to T0 in all soil layers (Fig. 5B–D), representing an increase between +0.3–0.4 (AL), +1.7–2.2 (TZ), and 1.6–2.0 (PF) in absolute abundance of gene copies per gram of soil (Log_10_ fold-change; Table S6). Genes associated with pyruvate metabolism increased significantly by T30 relative to T0, but only within the transition-zone and permafrost microbiomes (Fig. 5E), representing an increase of +0.9 (TZ) and +0.9 (PF) in absolute abundance of gene copies per gram of soil (Log_10_ fold-change; Table S6). Genes associated with methane metabolism decreased significantly by T30 relative to T0 in both the transition-zone and permafrost microbiomes (Fig. 5F), representing a decrease of −0.5 (TZ) and −0.3 (PF) in absolute abundance of gene copies per gram of soil (Log_10_ fold-change; Table S6).

## Discussion

Our laboratory incubation experiment simulating thaw conditions along the depth profile of an iron (Fe)-rich organic tundra soil revealed a substantial microbial response to extended (30-day) thaw duration in all soil layers that was driven primarily by the growth of bacterial taxa known to possess Fe-cycling capabilities. Gene abundance associated with Fe-cycling pathways and organic carbon (OC) degradation increased concurrently within all soil layers during the incubation, indicating a strong coupling between Fe(III) reduction, Fe(II) oxidation, and OC degradation following thaw. These findings suggest that microbial growth and activity during extended thaw was mediated primarily by the accessibility of reactive Fe under reducing conditions in the thawing tundra soil. Thus, Fe-mediated bacterial growth during extended permafrost thaw will likely impact the microbial oxidation of OC, simultaneously increasing CO_2_ production while suppressing CH_4_ production, in Fe-rich arctic tundra soils.

### Soil microbiome response to short-term thaw duration

The experimental results showed no significant microbiome response to a short-term thaw duration of 7 days (at 4 °C) in wet sedge tundra. The composition of the microbiome in all three soil layers remained unchanged after 7 days of thaw from their initial frozen composition (Fig. 2), with the transition-zone and permafrost microbiomes distinct from the active-layer microbiome yet indistinguishable from each other (Fig. 1A). Initial differences in taxonomic composition along the wet sedge tundra profile were consistent with our previous findings at this field site in northern Alaska [7], with greater relative abundance of Actinobacteriota, Caldisericota, and Firmicutes in the transition-zone and permafrost microbiomes (Fig. 2). There were also no significant changes in the relative abundance of Fe-associated functional genes (Fig. 4), normalized gene counts associated with OC degradation (Fig. 5), or any broader KEGG functional pathways (Fig. S4) annotated from the metagenomes following short-term thaw duration. These results suggest that short-term thaw duration is likely not capable of inducing a rapid shift in whole-community composition or gene abundance in any soil layer along the depth profile of wet sedge tundra, which is the dominant tundra type of arctic lowlands and coastal plains.

Yet previous studies documented significant and rapid shifts in the composition of the permafrost microbiome following short-term thaw durations [15–18]. In Mackleprang et al. [15], a short-term thaw duration of 2 days (at 5 °C) under reducing conditions caused the composition of the permafrost microbiome and its functional genes to shift rapidly to resemble the composition found in the overlying, seasonally thawed active-layer of a black-spruce forest site in sub-arctic Alaska. In Coolen and Orsi [16], a short-term thaw duration of 11 days (at 4 °C) under reducing conditions in moist acidic tussock tundra in the Imnavait Creek region caused the transcriptional profile of the permafrost microbiome to shift rapidly toward ribosomal protein production and enzymatic genes associated with OC degradation. However, more recent results from Barbato et al. [18] showed that medium-term thaw duration of 16 days (at 6 °C) caused a change in the permafrost microbiome composition and functional genes, but only in permafrost samples collected from certain locations, suggesting a differential response of permafrost microbes based on their origin.

The overall lack of a rapid microbial response to short-term thaw along the soil profile of wet sedge tundra (presented here) as compared to the substantial short-term microbial changes observed in thawing black spruce forest [15, 18] and moist acidic tussock tundra [16] is likely influenced by multiple factors including differences in dominant vegetation [64], substrate quality and quantity [22], and abiotic factors influencing initial composition of the microbiomes between sampling sites [65]. For example, the permafrost microbiome in wet sedge tundra was dominated by anaerobic and fermentative taxa belonging to Actinobacteriota, Caldisericota, and Firmicutes, where most of these taxa naturally dominate the microbiome under reducing conditions [66, 67] such that no shift in their abundance was observed after 7 days of thaw (Fig. 2). Findings from our recent study conducted, in part, at the same Imnavait Creek field site in northern Alaska also found that thaw duration is a strong environmental factor regulating the composition of the microbiome along the soil profile of wet sedge tundra [7]. Here, the short-term thaw duration of 7 days under reducing conditions may simply have been insufficient to allow the composition of the microbiome to shift.

### Soil microbiome response to extended thaw duration

An extended thaw duration of 30 days (at 4 °C incubation) was clearly sufficient to induce a microbiome-wide shift in taxonomic composition and functional gene abundance, especially within the transition-zone and permafrost microbiomes of wet sedge tundra (Figs. 1–5). These results support our previous findings that thaw duration is an important environmental factor regulating the composition of the active layer, transition zone, and permafrost microbiomes in wet sedge tundra [7]. Here, the growth of a single bacterial taxon, *Rhodoferax* sp. (most closely related to Fe(III)-reducing *Rhodoferax ferrireducens*), increased in both relative and absolute abundance during extended thaw to become the most dominant taxon in each soil layer microbiome (Table 1). The absolute abundance of this *Rhodoferax* sp. increased by 2-orders of magnitude in the active-layer microbiome and by more than 4-orders of magnitude in the transition-zone and permafrost microbiomes following 30 days of thaw (Fig. 3A). Emerson et al. [68] previously found that *R. ferrireducens* was the most abundant taxon recovered (>50% of total reads) from numerous microbial iron mats occurring in the Imnavait Creek region, suggesting a dominant role for this Fe(III)-reducing taxon in Fe-rich tundra surface soils. Our results indicate that this organism can also dominate in deeper, Fe-rich soils following an extended thaw duration. *R. ferrireducens* is a facultative Fe(III)-reducing anaerobe capable of dissimilatory Fe(III) reduction when coupled with OC degradation via acetate and benzoate oxidation to CO_2_ [69]. As such, we found a concurrent increase in the relative abundance of genes associated with Fe(III) reduction (Fig. 4E), benzoate, aminobenzoate, and fluorobenzoate degradation (Fig. 5B–D), and pyruvate metabolism (Fig. 5E) after 30 days of thaw, especially within the transition-zone and permafrost microbiomes. These results indicate strong coupling between Fe(III) reduction and the degradation of OC by *Rhodoferax* sp. following extended permafrost thaw.

We also found that *Gallionella* sp., a microaerophilic Fe(II)-oxidizing chemoautotroph [70, 71], increased by ~8% in relative abundance in the active-layer microbiome and by ~2–3% in the transition-zone and permafrost microbiomes (Table 1). This was also equivalent to an increase of 3–5 orders of magnitude in the absolute abundance of *Gallionella* 16S gene copies in each soil layer after 30 days of thaw, with absolute growth rates similar to those observed for *Rhodoferax* sp. (Fig. 3). *Gallionella* spp. have previously been observed in arctic ponds [68] and palsa hillslopes [36] and are capable of oxidizing Fe(II) using CO_2_ as an electron acceptor, where CO_2_ is reduced back to acetate [72]. These Fe-cycling metabolic processes between *Rhodoferax* sp. and *Gallionella* sp. appear to be complementary to each other, where the heterotrophic *Rhodoferax* sp. oxidizes acetate and benzoate to CO_2_ to facilitate Fe(III) reduction while the chemoautotrophic *Gallionella* sp. fixes CO_2_ back to acetate when oxidizing Fe(II) with oxygen. It is well known that the oxidation and reduction of iron occurs cyclically in many environments [73]. Thus, this response likely represents a syntrophic relationship between *Rhodoferax* sp. and *Gallionella* sp., where each species consumes the metabolic products of the other species to perpetuate their substantial Fe-mediated growth during extended duration of permafrost thaw.

We note, however, that the Fe-cycling metabolic processes facilitating *Rhodoferax* and *Gallionella* growth assume that reducing conditions during the thaw experiment were sub-oxic (0.1–1% O_2_ concentration) rather than strictly anoxic. This is because the microaerophilic *Gallionella* sp. requires sub-oxic conditions to support its growth (0.1–1% O_2_ concentration) [74]. Meanwhile, *R. ferrireducens* is one of only two known facultative Fe(III)-reducing anaerobes capable of reducing Fe(III) under sub-oxic conditions [69]. Further support for assuming sub-oxic rather than anoxic conditions comes from the lack of growth of *Geobacter* sp. (Desulfobacterota) throughout the experiment (<1% relative abundance; Table 1). *Geobacter* is a strictly obligate Fe(III)-reducing anaerobe [75] and typically the dominant Fe(III)-reducer in the anoxic zone of Fe-cycling biofilms in tundra soils [31, 68]. The lack of growth of *Geobacter* sp. coupled with the substantial growth of *Rhodoferax* sp. and *Gallionella* sp. suggests that the incubation conditions were sub-oxic and not anoxic. The presence of very small amounts of oxygen may be common in low-lying, wet sedge tundra soils, where oxygen may be supplied by a variety of processes including through plant aerenchyma and hydrological inputs [76].

Numerous studies have already shown that permafrost thaw exposes previously frozen tundra soils to reducing conditions that favor the reductive dissolution of reactive Fe minerals [34, 36, 77]. Here, we show that the Fe(III)-reducing bacterium, *Rhodoferax* sp., uses reactive Fe(III) for anaerobic respiration when coupled with OC, likely promoting CO_2_ formation following permafrost thaw. Furthermore, the oxidation of acetate by *Rhodoferax* sp. when coupled with Fe(III) reduction can limit acetate availability for methanogenesis via the acetoclastic pathway, which is a major methanogenic pathway in organic tundra soils [78]. Consistent with this idea, our results show a significant decrease in relative gene abundance annotated for CH_4_ metabolism following extended thaw duration (Fig. 5F), mostly associated with methanogenic pathways annotated to archaeal species known for acetoclastic methanogenesis including *Methanothrix* and *Methanosarcina* spp. [79, 80]. These results are also consistent with recent studies investigating *in situ* rates and pathways of Fe(III)-reduction in thawing permafrost hillslopes, where substantial growth of *Rhodoferax* sp. and other Fe(III)-reducing taxa promoted greater gene expression for dissimilatory Fe(III) reduction that suppressed acetoclastic methanogenesis [36]. This response occurs because dissimilatory Fe(III) reduction is more thermodynamically (energy) favorable than methanogenesis under reducing conditions [34]. Thus, there will likely be an increased ratio of CO_2_ production to CH_4_ production in Fe-rich permafrost soils following extended thaw duration. It is possible that this effect may be persistent in iron-rich soils, in part due to the amount of iron available and in part due to the regenerative nature of iron redox cycling.

## Conclusion

Results from this study show that an extended thaw duration of 30 days promotes the growth of Gammaproteobacteria (+2 to +5 orders of magnitude in absolute abundance), specifically the heterotrophic Fe(III)-reducing *Rhodoferax* sp. and chemoautotrophic Fe(II)-oxidizing *Gallionella* sp., especially within the transition-zone and permafrost microbiomes. The active-layer microbiome experienced a similar shift in composition with an increase in Fe-cycling taxa, but the magnitude of this shift was more limited (+20% increase in relative abundance) compared to shifts in the transition-zone and permafrost microbiomes (+65%, each). Our results suggest that dissolved Fe(III) supports the growth of Fe-cycling taxa by providing a terminal electron acceptor for pathways of anaerobic respiration associated with the degradation of aromatic compounds such as acetate and benzoate, leading to CO_2_ production while also suppressing CH_4_ metabolism and production. As thaw duration increases at depth due to climate warming, it is likely that Fe-mediated microbial respiration of organic carbon will play a dominant role in the permafrost carbon cycle of wet sedge tundra. The immediate implications of these findings apply broadly across the Arctic because wet sedge tundra constitutes the major tundra type of low-lying regions along the foothills and coastal plains.

### Supplementary information

Supplemental Materials.

Supplemental Dataset

## Data Availability

Raw read sequences for 16S rRNA amplicons and metagenomes were submitted to the NCBI Sequence Read Archive under BioProject accession PRJNA976224.
