# Supplementary material for: Genomic evidence that microbial carbon degradation is dominated by iron redox metabolism in thawing permafrost
Source: ISME Commun. 2023 Nov 23;3:124. doi: 10.1038/s43705-023-00326-5 (PMC10667234; doi:10.1038/s43705-023-00326-5)
Supplement: Supplementary file 1 — Supplemental Materials. [file 43705_2023_326_MOESM1_ESM.pdf]

Supplemental Materials

Genomic evidence that microbial carbon degradation is dominated by iron redox metabolism in thawing permafrost

Karl J. Romanowicz [1], Byron C. Crump [2], George W. Kling [1*]

[1] Department of Ecology and Evolutionary Biology, University of Michigan, Ann Arbor, Michigan, USA
[2] College of Earth, Ocean, and Atmospheric Sciences, Oregon State University, Corvallis, Oregon, USA
*** Correspondence:** George W. Kling (gwk@umich.edu)

Absolute abundance of 16S rRNA genes: To determine the number of 16S rRNA genes found in each extracted soil sample, we used the following equation described in Smets et al. (2016):

$$\frac{R_i}{R_s} = \frac{\frac{w_i}{g_i} \times c_i}{X} \Longrightarrow X = \frac{R_s \times \left(\frac{w_i}{g_i} \times c_i\right)}{R_i} \quad \text{[Eq. 1]}$$

where R_i is the number of reads assigned to the bacterial taxon used as the internal standard (*Thermus thermophilus*), R_s is the number of reads assigned to the other taxa that were found in the soil, w_i is the weight of the internal standard gDNA added to the samples (5.5 ng *T. thermophilus* per gram soil), g_i is the weight of the genome of the internal standard (2.16 x 10^{-15} g), c_i is the 16S copy number of the internal standard (2 copies), and X is the (initially unknown) number of 16S rRNA gene copies per gram of soil per sample. The DNA of the internal standard was added before DNA extraction so that if DNA extraction yields varied between samples, such variation would not interfere with this calculation assuming that the recovery of DNA from the internal standard and the recovery of DNA from the native bacterial cells is similar.

Absolute abundance of KEGG ortholog genes per gram of soil: To determine the number of KEGG ortholog (KO) genes found in each extracted soil sample from the metagenome sequencing data, we followed methods described in Baker et al. (2021). Briefly, paired-end sequence reads were quality trimmed (BBDuk v.38.84; Bushnell 2014), and internal *T. thermophilus* standard sequences were identified in a two-step process using BBSplit (BBTools; Bushnell 2014) with a reference database of the *T. thermophilus* HB-8 genome, and with Kaiju (Menzel et al. 2016) with a reference database built using RefSeq (O'Leary et al. 2016). *T. thermophilus* reads were removed from the dataset, and the remaining reads for each sample were then assembled individually with metaSPAdes using default settings (v 3.11.0; Nurk et al. 2017). *T. thermophilus* sequences in each sample were enumerated (forward + reverse) and used to determine multipliers to calculate genes per gram soil. Briefly, the multipliers (T_t) for each sample were calculated as:

$$T_t = \frac{S_a}{(S_r \times gram\ soil)} \quad \text{[Eq. 2]}$$

where S_a is the number of molecules of *T. thermophilus* genomes added to the sample, and S_r is the number of *T. thermophilus* genomes recovered. S_r was calculated by dividing the number of *T. thermophilus* sequences recovered by the number of genes in the *T. thermophilus* genome (2,173).

Contig files for all samples were concatenated and dereplicated, and contigs less than 200 nucleotides in length were removed. Concatenated contig sequences were submitted to IMG-

MER for annotation with the DOE-JGI Metagenome Annotation Pipeline (Huntemann et al. 2016). After annotation, coding sequences (i.e., CDS or sequences coding for genes) were extracted from contigs to produce a Bowtie2 database of CDS sequences (Langmead & Salzberg 2012). The quality-controlled sequences that were used for assembly (not including *T. thermophilus* sequences) were mapped to this database, and read counts were divided into separate files based on domain (Bacteria, Archaea, Eukaryote, Virus). For this study, only the Bacteria and Archaea sequences were further analyzed. Read counts for each CDS were normalized following Wagner et al. (2012) for relative abundance and were also used to calculate absolute gene abundance following Satinsky et al. (2013). Briefly, the read count for each CDS was corrected for gene length and read length. To calculate genes per million (GPM) relative abundance for each CDS, this corrected read count (T_g) was scaled to 1 million:

$$GPM = T_g \times \left(1 \times 10^6 \,/\, \Sigma T_g\right) \quad \text{[Eq. 3]}$$

Genes per gram soil absolute abundance (T_g) was scaled to the original sum of mapped reads:

$$T_{g\_scaled} = T_g \times \left(\Sigma R_g \,/\, \Sigma T_g\right) \quad \text{[Eq. 4]}$$

and then multiplied by the *T. thermophilus* multiplier (T_t) for that sample (see Eq. 2 for T_t):

$$T_{g\_abs} = T_{g\_scaled} \times T_t \quad \text{[Eq. 5]}$$

We summed abundances for CDS assigned to the same KO number for each abundance measure.

References

Baker, K.D., Kellogg, C.T.E., McClelland, J.W., et al. (2021). The genomic capabilities of microbial communities to track seasonal variation in environmental conditions of arctic lagoons. Front. Microbiol. 12:601901.

Bushnell, B. (2014). BBMap: A Fast, Accurate, Splice-Aware Aligner (No. LBNL- 7065E). Berkeley, CA: Lawrence Berkeley National Lab (LBNL).

Huntemann, M., Ivanova, N. N., Mavromatis, K., et al. (2016). The standard operating procedure of the DOE- JGI Metagenome Annotation Pipeline. Stand. Genomic Sci. 11:17.

Langmead, B., and Salzberg, S. L. (2012). Fast gapped-read alignment with Bowtie 2. Nat. Methods 9, 357–359.

Menzel, P., Ng, K. L., and Krogh, A. (2016). Fast and sensitive taxonomic classification for metagenomics with Kaiju. Nat. Commun. 7:11257.

Nurk, S., Meleshko, D., Korobeynikov, A., and Pevzner, P. A. (2017). metaSPAdes: a new versatile metagenomic assembler. Genome Res. 27, 824–834.

O'Leary, N. A., Wright, M. W., Brister, J. R., et al. (2016). Reference sequence (RefSeq) database at NCBI: current status, taxonomic expansion, and functional annotation. Nucleic Acids Res. 44, D733– D745.

Satinsky, B. M., Gifford, S. M., Crump, B. C., and Moran, M. A. (2013). "Use of internal standards for quantitative metatranscriptome and metagenome analysis," in Methods in Enzymology, eds J. Abelson, M. Simon, G. Verdine, and A. Pyle (Amsterdam: Elsevier), 237–250.

Smets, W., Leff, J.W., Bradford, M.A., et al. (2016). A method for simultaneous measurement of soil bacterial abundances and community composition via 16S rRNA gene sequencing. Soil Biol. Biochem. 96, 145-151.

Wagner, G. P., Kin, K., and Lynch, V. J. (2012). Measurement of mRNA abundance using RNA-seq data: RPKM measure is inconsistent among samples. Theory Biosci. 131, 281–285.

Table S1. Summary of 16S rRNA amplicon (DNA) sequence reads with alpha diversity metrics. The sample with the lowest total reads is denoted in **bold** and represents the read rarefaction cutoff value used for the entire dataset.

Sample ID	Soil Layer	Time Point	Incubation Replicate	Total Reads	Rarified Reads	ASV Unique	ASV Diversity (H')
AL.T0.1	AL	T0	1	200,301	71,245	1,084	4.91
AL.T0.2	AL	T0	2	317,272	71,245	2,261	5.87
AL.T0.3	AL	T0	3	409,943	71,245	2,534	5.99
AL.T7.1	AL	T7	1	329,477	71,245	1,461	5.43
AL.T7.2	AL	T7	2	342,483	71,245	1,211	5.07
AL.T7.3	AL	T7	3	390,248	71,245	1,474	5.33
AL.T30.1	AL	T30	1	216,755	71,245	1,001	4.81
AL.T30.2	AL	T30	2	280,778	71,245	1,003	4.66
AL.T30.3	AL	T30	3	252,338	71,245	1,068	4.71
TZ.T0.1	TZ	T0	1	208,767	71,245	957	4.21
TZ.T0.2	TZ	T0	2	182,419	71,245	676	3.71
TZ.T0.3	TZ	T0	3	214,475	71,245	949	4.27
TZ.T7.1	TZ	T7	1	227,446	71,245	803	3.83
TZ.T7.2	TZ	T7	2	220,625	71,245	878	4.10
TZ.T7.3	TZ	T7	3	247,654	71,245	1,033	4.28
TZ.T30.1	TZ	T30	1	99,212	71,245	80	1.07
TZ.T30.2	TZ	T30	2	118,575	71,245	92	1.45
TZ.T30.3	TZ	T30	3	151,341	71,245	95	1.56
PF.T0.1	PF	T0	1	247,542	71,245	1,028	4.48
PF.T0.2	PF	T0	2	239,184	71,245	1,038	4.56
PF.T0.3	PF	T0	3	243,173	71,245	987	4.34
PF.T7.1	PF	T7	1	231,466	71,245	1,127	4.73
PF.T7.2	PF	T7	2	272,013	71,245	1,431	4.94
PF.T7.3	PF	T7	3	255,596	71,245	1,075	4.52
PF.T30.1	PF	T30	1	113,760	71,245	146	1.85
**PF.T30.2**	**PF**	**T30**	**2**	**71,245**	**71,245**	**103**	**1.46**
PF.T30.3	PF	T30	3	105,537	71,245	133	1.42

Table S2. Summary of metagenome (DNA) sequence reads with alpha diversity metrics.

Sample ID	Soil Layer	Time Point	Incubation Replicate	Mapped Reads	Proper Pairs (%)	Mean Coverage (%)	Gene Richness	Gene Abundance	Gene Diversity (H')
MG37	AL	T0	1	7,306,878	54.1	41.4	442,009	2,105,340	12.30
MG38	AL	T0	2	4,952,240	51.8	28.0	372,252	1,423,614	12.23
MG39	AL	T0	3	5,505,102	46.1	31.1	396,670	1,575,218	12.23
MG46	AL	T7	1	7,964,504	39.4	45.1	502,644	2,270,238	12.40
MG47	AL	T7	2	7,661,574	46.0	43.4	469,720	2,187,108	12.34
MG48	AL	T7	3	7,086,521	44.2	40.2	455,828	2,017,807	12.31
MG55	AL	T30	1	7,523,832	31.2	42.5	521,939	2,267,908	12.36
MG56	AL	T30	2	8,837,029	39.6	50.0	533,492	2,682,524	12.34
MG57	AL	T30	3	8,503,122	44.4	47.9	530,499	2,581,525	12.41
MG40	TZ	T0	1	6,652,912	52.3	37.5	245,198	2,146,489	10.52
MG41	TZ	T0	2	6,796,127	70.2	38.4	184,410	2,227,399	10.35
MG42	TZ	T0	3	7,032,841	58.8	39.5	219,975	2,285,000	10.58
MG49	TZ	T7	1	10,892,048	39.0	61.5	237,430	3,569,391	10.09
MG50	TZ	T7	2	6,731,231	48.0	37.9	218,567	2,166,288	10.60
MG51	TZ	T7	3	7,796,826	61.5	44.0	215,057	2,490,079	10.47
MG58	TZ	T30	1	9,614,409	33.4	54.5	153,088	3,455,362	9.27
MG59	TZ	T30	2	11,002,154	42.5	62.3	182,586	3,906,642	9.51
MG60	TZ	T30	3	9,780,558	44.9	55.4	176,793	3,510,015	9.93
MG43	PF	T0	1	6,815,432	53.7	38.3	212,433	2,201,935	10.65
MG44	PF	T0	2	6,378,144	50.6	35.9	206,848	2,085,707	10.56
MG45	PF	T0	3	8,018,779	49.9	45.2	223,769	2,636,855	10.50
MG52	PF	T7	1	8,347,188	33.9	47.0	249,708	2,652,600	10.74
MG53	PF	T7	2	8,664,363	54.1	48.7	265,488	2,729,196	10.96
MG54	PF	T7	3	8,161,837	41.9	45.9	235,465	2,590,998	10.68
MG61	PF	T30	1	10,025,330	41.0	56.7	205,693	3,552,264	10.05
MG62	PF	T30	2	10,390,831	40.7	58.8	192,847	3,798,916	9.77
MG63	PF	T30	3	10,113,057	35.1	57.3	180,851	3,604,546	9.70

Table S3. Relative abundance (mean % ± SD) of dominant bacterial phyla and archaea derived from 16S rRNA amplicon sequence variants (ASVs). Proteobacteria are resolved to class. Percent change values denote the mean change in relative abundance for each taxon compared to incubation time point T0 within each soil layer microbiome. **Bold** values indicate a significant difference in mean relative abundance for each taxon compared to incubation time point T0 within each soil layer microbiome (ANOVA; $p < 0.05$).

Soil Layer & Time Point	Acidobacteriota		Actinobacteriota		Bacteroidota		Caldisericota		Chloroflexi		Deinococcota		Desulfobacterota	
	Rel. Abund. (%)	% Change from T0	Rel. Abund. (%)	% Change from T0	Rel. Abund. (%)	% Change from T0	Rel. Abund. (%)	% Change from T0	Rel. Abund. (%)	% Change from T0	Rel. Abund. (%)	% Change from T0	Rel. Abund. (%)	% Change from T0
**Active Layer**														
T0	10.6 ± 3.3	-	7.5 ± 1.0	-	28.7 ± 11.3	-	1.5 ± 0.5	-	8.2 ± 1.6	-	< 0.1	-	5.1 ± 0.5	-
T7	6.9 ± 0.5	-3.7	9.5 ± 1.4	2.0	31.1 ± 2.9	2.4	1.9 ± 0.3	0.4	7.4 ± 0.7	-0.8	0.3 ± 0.2	0.3	6.5 ± 0.6	1.4
T30	6.3 ± 0.8	-4.3	7.7 ± 0.4	0.2	23.6 ± 3.5	-5.1	1.3 ± 0.4	-0.2	**4.8 ± 1.2**	**-3.4**	< 0.1	0.0	5.8 ± 0.9	0.7
**Transition Zone**														
T0	3.7 ± 0.8	-	31.3 ± 4.9	-	4.7 ± 1.8	-	28.4 ± 4.8	-	6.1 ± 1.7	-	4.2 ± 0.2	-	3.4 ± 0.4	-
T7	4.1 ± 0.3	0.4	28.4 ± 3.1	-2.9	4.3 ± 0.5	-0.4	31.5 ± 5.9	3.1	6.4 ± 0.8	0.3	5.3 ± 2.0	1.1	3.9 ± 0.9	0.5
T30	**< 0.1**	**-3.7**	**2.0 ± 0.8**	**-29.3**	**0.2 ± 0.1**	**-4.5**	**0.7 ± 0.2**	**-27.7**	**< 0.1**	**-6.1**	**0.2 ± 0.1**	**-4.0**	**< 0.1**	**-3.4**
**Permafrost**														
T0	4.8 ± 0.3	-	33.3 ± 0.7	-	6.2 ± 0.2	-	18.6 ± 2.6	-	8.1 ± 0.6	-	3.0 ± 0.6	-	5.3 ± 1.1	-
T7	5.6 ± 0.1	0.8	31.5 ± 1.7	-1.8	7.3 ± 0.6	1.1	16.1 ± 2.9	-2.5	10.6 ± 2.2	2.5	4.3 ± 1.0	1.3	4.2 ± 0.5	-1.1
T30	**< 0.1**	**-4.8**	**2.1 ± 0.6**	**-31.2**	**0.6 ± 0.3**	**-5.6**	**0.5 ± 0.1**	**-18.1**	**0.2 ± 0.1**	**-7.9**	**< 0.1**	**-3.0**	**0.2 ± 0.1**	**-5.1**

Soil Layer & Time Point	Firmicutes		Verrucomicrobiota		Alphaproteobacteria		Gammaproteobacteria		Bacteria Other		Archaea	
	Rel. Abund. (%)	% Change from T0	Rel. Abund. (%)	% Change from T0	Rel. Abund. (%)	% Change from T0	Rel. Abund. (%)	% Change from T0	Rel. Abund. (%)	% Change from T0	Rel. Abund. (%)	% Change from T0
**Active Layer**												
T0	1.5 ± 0.2	-	15.5 ± 0.9	-	4.7 ± 2.3	-	2.9 ± 1.7	-	13.2 ± 3.4	-	0.6 ± 0.1	-
T7	2.4 ± 0.5	0.9	15.7 ± 1.4	0.2	2.7 ± 0.3	-2.0	2.1 ± 0.7	-0.8	12.6 ± 2.1	-0.6	0.9 ± 0.1	0.3
T30	1.5 ± 0.4	0.0	**10.1 ± 1.5**	**-5.4**	2.0 ± 0.3	-2.7	**28.7 ± 2.2**	**25.8**	7.8 ± 1.0	-5.4	0.5 ± 0.1	-0.1
**Transition Zone**												
T0	10.6 ± 0.7	-	1.1 ± 0.9	-	0.7 ± 0.2	-	0.4 ± 0.2	-	4.9 ± 0.7	-	0.4 ± 0.1	-
T7	8.4 ± 0.4	-2.2	0.7 ± 0.1	-0.4	0.9 ± 0.1	0.2	0.5 ± 0.1	0.1	4.9 ± 1.1	0.0	0.6 ± 0.1	0.2
T30	13.5 ± 4.6	2.9	< 0.1	-1.1	**1.2 ± 0.1**	**0.5**	**81.9 ± 5.3**	**81.5**	**< 0.1**	**-4.9**	**< 0.1**	**-0.4**
**Permafrost**												
T0	11.0 ± 0.9	-	1.0 ± 0.1	-	0.9 ± 0.1	-	0.5 ± 0.2	-	6.6 ± 0.4	-	0.6 ± 0.1	-
T7	8.6 ± 0.4	-2.4	0.9 ± 0.2	-0.1	1.3 ± 0.2	0.4	0.7 ± 0.4	0.2	7.8 ± 0.8	1.2	0.9 ± 0.2	0.3
T30	7.5 ± 0.4	-3.5	**< 0.1**	**-1.0**	1.0 ± 1.4	0.1	**87.5 ± 2.0**	**87.0**	**0.2 ± 0.1**	**-6.4**	**< 0.1**	**-0.6**

Table S4. Absolute abundance (mean 16S rRNA gene copies) for dominant bacterial phyla and archaea derived from 16S rRNA amplicon sequencing relative to *Thermus thermophilus* internal standard recovered from sequencing. Proteobacteria are resolved to class. Log fold-change (LogFC using log10) values denote the mean magnitude of change compared to incubation time point T0 within each soil layer microbiome. **Bold** values indicate a significant difference in mean 16S rRNA gene copies for each taxon compared to incubation time point T0 within each soil layer microbiome (ANOVA; $p < 0.05$).

Soil Layer & Time Point	Acidobacteriota		Actinobacteriota		Bacteroidota		Caldisericota		Chloroflexi		Deinococcota		Desulfobacterota	
	16S Gene Copy Abund.	LogFC from T0	16S Gene Copy Abund.	LogFC from T0	16S Gene Copy Abund.	LogFC from T0	16S Gene Copy Abund.	LogFC from T0	16S Gene Copy Abund.	LogFC from T0	16S Gene Copy Abund.	LogFC from T0	16S Gene Copy Abund.	LogFC from T0
**Active Layer**														
T0	1,492,021,353	-	1,079,340,985	-	4,451,642,750	-	230,667,248	-	1,176,405,157	-	19,780,481	-	756,400,010	-
T7	642,572,575	-0.37	844,467,215	-0.11	2,725,707,580	-0.21	168,641,097	-0.14	686,746,134	-0.23	19,844,840	0.00	565,460,268	-0.13
T30	1,492,788,901	0.00	1,849,187,045	0.23	6,009,879,015	0.13	317,332,858	0.14	1,077,275,985	-0.04	19,784,836	0.00	1,505,354,571	0.30
**Transition Zone**														
T0	15,367,478	-	127,520,603	-	19,414,831	-	115,136,590	-	25,100,227	-	17,062,092	-	13,787,088	-
T7	17,928,276	0.07	121,790,295	-0.02	22,266,237	0.06	67,365,157	-0.23	30,369,705	0.08	11,140,765	-0.19	18,478,203	0.13
T30	6,188,812	-0.39	220,191,046	0.24	17,172,486	-0.05	71,069,500	-0.21	10,130,042	-0.39	17,778,488	0.02	9,867,187	-0.15
**Permafrost**														
T0	29,184,935	-	206,635,802	-	30,721,799	-	227,281,274	-	45,817,651	-	38,973,541	-	29,178,041	-
T7	28,621,464	-0.01	159,232,504	-0.11	37,346,592	0.08	81,730,558	-0.44	54,366,154	0.07	20,939,218	-0.27	21,797,074	-0.13
T30	30,975,186	0.03	497,958,366	0.38	128,172,426	0.62	**101,378,798**	**-0.35**	55,698,678	0.08	21,856,156	-0.25	38,587,531	0.12

Soil Layer & Time Point	Firmicutes		Verrucomicrobiota		Alphaproteobacteria		Gammaproteobacteria		Bacteria Other		Archaea	
	16S Gene Copy Abund.	LogFC from T0	16S Gene Copy Abund.	LogFC from T0	16S Gene Copy Abund.	LogFC from T0	16S Gene Copy Abund.	LogFC from T0	16S Gene Copy Abund.	LogFC from T0	16S Gene Copy Abund.	LogFC from T0
**Active Layer**												
T0	218,437,400	-	2,289,946,358	-	650,841,187	-	399,279,553	-	1,857,428,134	-	85,951,823	-
T7	226,001,157	0.01	1,446,585,269	-0.20	237,775,105	-0.44	194,124,319	-0.31	1,196,111,807	-0.19	83,539,493	-0.01
T30	396,521,913	0.26	2,445,814,831	0.03	463,495,608	-0.15	7,136,589,672	1.25	1,816,065,248	-0.01	123,129,027	0.16
**Transition Zone**												
T0	43,188,717	-	4,554,561	-	2,884,710	-	1,846,043	-	19,877,831	-	1,748,850	-
T7	39,921,797	-0.03	3,529,818	-0.11	3,224,829	0.05	2,096,898	0.06	23,923,944	0.08	2,371,429	0.13
T30	**1,401,438,564**	**1.51**	1,735,416	-0.42	**124,015,978**	**1.63**	**8,737,065,010**	**3.68**	5,259,542	-0.58	461,542	-0.58
**Permafrost**												
T0	60,996,116	-	4,697,323	-	6,570,124	-	3,637,120	-	34,710,315	-	4,229,848	-
T7	43,889,372	-0.14	4,676,808	0.00	6,599,175	0.00	3,619,556	0.00	40,071,210	0.06	4,402,246	0.02
T30	**1,701,273,905**	**1.45**	7,690,064	0.21	**259,035,158**	**1.60**	**19,696,798,643**	**3.73**	37,824,439	0.04	3,110,952	-0.13

Table S5. Absolute abundance (mean) of 16S rRNA genes and functional genes associated with Fe(III)-reducing and Fe(II)-oxidizing bacterial taxa derived from the 16S rRNA amplicon sequencing and metagenome sequencing, respectively, relative to *Thermus thermophilus* internal standard recovered from each sequencing effort. Log fold-change (LogFC using log10) values denote the mean magnitude of change compared to incubation time point T0 within each soil layer microbiome. **Bold** values indicate a significant difference in mean 16S rRNA gene copies or mean functional gene copies for each Fe-related taxon compared to incubation time point T0 within each soil layer microbiome (ANOVA; $p < 0.05$).

Soil Layer & Timepoint	Fe(III)-Reduction								Fe(II)-Oxidation			
	Rhodoferax sp.				*Geobacter* sp.				*Gallionella* sp.			
	16S Gene Copy Abund.	LogFC from T0	Function Genes $Gram^{-1}$ Soil	LogFC from T0	16S Gene Copy Abund.	LogFC from T0	Function Genes $Gram^{-1}$ Soil	LogFC from T0	16S Gene Copy Abund.	LogFC from T0	Function Genes $Gram^{-1}$ Soil	LogFC from T0
**Active Layer**												
T0	2.1E+07	-	6.5E+10	-	2.7E+07	-	1.2E+10	-	1.0E+07	-	3.9E+10	-
T7	5.2E+07	0.39	1.3E+11	0.30	3.0E+07	0.04	1.1E+10	-0.02	2.4E+07	0.38	3.4E+10	-0.06
T30	**2.6E+09**	**2.09**	**2.1E+12**	**1.51**	2.7E+08	0.99	1.2E+10	0.00	**1.7E+09**	**2.24**	**8.7E+11**	**1.35**
**Transition Zone**												
T0	2.3E+05	-	1.4E+09	-	2.5E+05	-	1.1E+09	-	3.7E+04	-	6.2E+08	-
T7	4.1E+05	0.24	9.4E+08	-0.19	1.3E+05	-0.29	1.1E+09	-0.01	3.3E+04	-0.05	4.3E+08	-0.15
T30	**6.5E+09**	**4.44**	**7.0E+12**	**3.69**	5.9E+05	0.38	1.0E+09	-0.03	**1.6E+08**	**3.63**	**1.8E+11**	**2.47**
**Permafrost**												
T0	3.8E+05	-	1.9E+09	-	3.4E+05	-	2.4E+09	-	5.1E+04	-	1.2E+09	-
T7	3.8E+05	0.00	1.3E+09	-0.18	2.6E+05	-0.11	1.2E+09	-0.31	4.1E+04	-0.09	5.7E+08	-0.32
T30	**1.4E+10**	**4.57**	**1.2E+13**	**3.78**	2.2E+05	-0.19	2.3E+09	-0.01	**6.2E+08**	**4.08**	**4.6E+11**	**2.59**

Table S6. Absolute abundance (mean) of KO genes gram^{-1} soil associated with KEGG Tier IV metabolism pathways related to the degradation of aromatic compounds, pyruvate metabolism, and methane metabolism derived from the metagenome sequencing relative to *Thermus thermophilus* internal standard recovered from sequencing. Log fold-change (LogFC using log10) values denote the mean magnitude of change in KO gene counts compared to incubation time point T0 within each soil layer microbiome. **Bold** values indicate a significant difference in mean counts for each KEGG pathway compared to incubation time point T0 within each soil layer microbiome (ANOVA; $p < 0.05$).

Soil Layer & Time Point	Aromatic Compound Degradation		Benzoate Degradation		Aminobenzoate Degradation		Fluorobenzoate Degradation		Pyruvate Metabolism		Methane Metabolism	
	Genes gram^{-1} soil	LogFC from T0	Genes gram^{-1} soil	LogFC from T0	Genes gram^{-1} soil	LogFC from T0	Genes gram^{-1} soil	LogFC from T0	Genes gram^{-1} soil	LogFC from T0	Genes gram^{-1} soil	LogFC from T0
**Active Layer**												
T0	4.8E+08	-	5.0E+08	-	1.5E+08	-	1.1E+10	-	2.1E+09	-	9.6E+08	-
T7	4.7E+08	0.0	4.7E+08	0.0	1.4E+08	0.0	1.1E+10	0.0	2.3E+09	0.0	6.6E+08	-0.2
T30	**9.0E+08**	**0.3**	**1.3E+09**	**0.4**	**3.5E+08**	**0.4**	**2.0E+10**	**0.3**	3.1E+09	0.2	6.1E+08	-0.2
**Transition Zone**												
T0	2.9E+07	-	1.1E+07	-	6.4E+06	-	2.9E+08	-	2.6E+08	-	**2.7E+08**	-
T7	2.3E+07	-0.1	7.2E+06	-0.2	5.0E+06	-0.1	2.2E+08	-0.1	2.3E+08	-0.1	6.3E+07	-0.6
T30	**1.0E+09**	**1.6**	**1.7E+09**	**2.2**	**3.1E+08**	**1.7**	**1.7E+10**	**1.8**	**2.0E+09**	**0.9**	8.2E+07	**-0.5**
**Permafrost**												
T0	6.9E+07	-	2.9E+07	-	1.4E+07	-	5.9E+08	-	5.5E+08	-	**4.3E+08**	-
T7	3.3E+07	-0.3	1.0E+07	-0.4	1.0E+07	-0.1	3.2E+08	-0.3	2.6E+08	-0.3	1.2E+08	-0.6
T30	**1.7E+09**	**1.4**	**2.9E+09**	**2.0**	**5.0E+08**	**1.6**	**3.1E+10**	**1.7**	**3.6E+09**	**0.8**	2.0E+08	**-0.3**

Table S7. List of the number of unique protein families incorporated in hidden Markov models (HMMs) in FeGenie for each Fe-associated category.

Fe-Associated Categories in FeGenie	Protein Families
Fe Regulation	7
Fe Storage	3
Fe Transport	10
Siderophore Production	18
Fe(III) Reduction	13
Fe(II) Oxidation	8

Table S8. List of the number of unique genes included in KEGG tier IV categories related to the degradation of organic carbon. See Supplemental Dataset for a full list of gene annotations within each KO category and their relative and absolute abundance values.

KEGG Tier IV Category	Unique Genes
01220 Degradation of aromatic compounds [PATH:ko01220]	197
00362 Benzoate degradation [PATH:ko00362]	28
00627 Aminobenzoate degradation [PATH:ko00627]	37
00364 Fluorobenzoate degradation [PATH:ko00364]	6
00620 Pyruvate metabolism [PATH:ko00620]	31
00680 Methane metabolism [PATH:ko00680]	45

SUPPLEMENTAL FIGURES

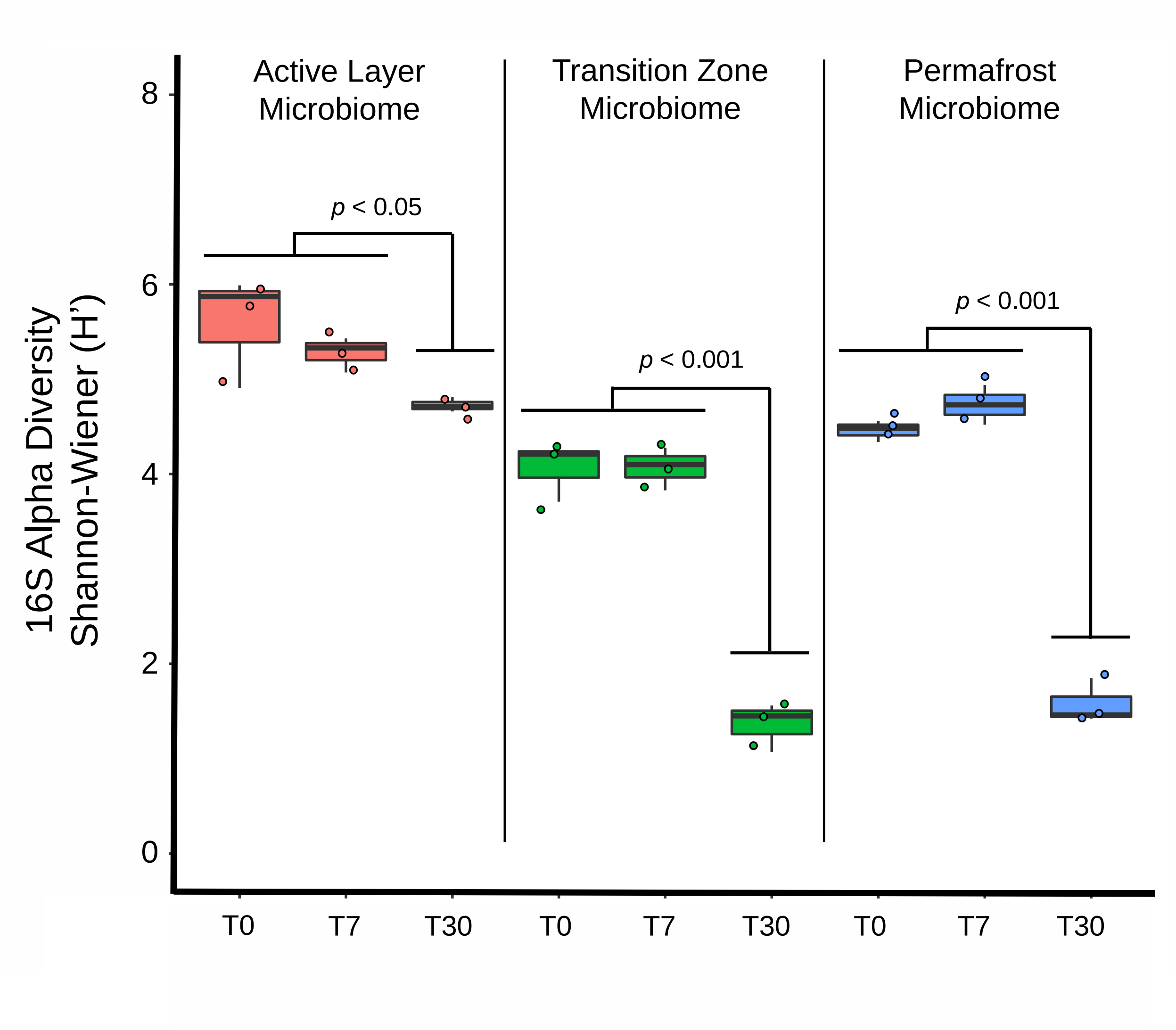

Fig. S1. Boxplot representing the alpha diversity (mean H' ± SD) of 16S rRNA amplicon sequence variants (ASVs) based on the Shannon diversity metric (H'). Significant differences were determined between incubation time points within each soil layer microbiome (ANOVA).

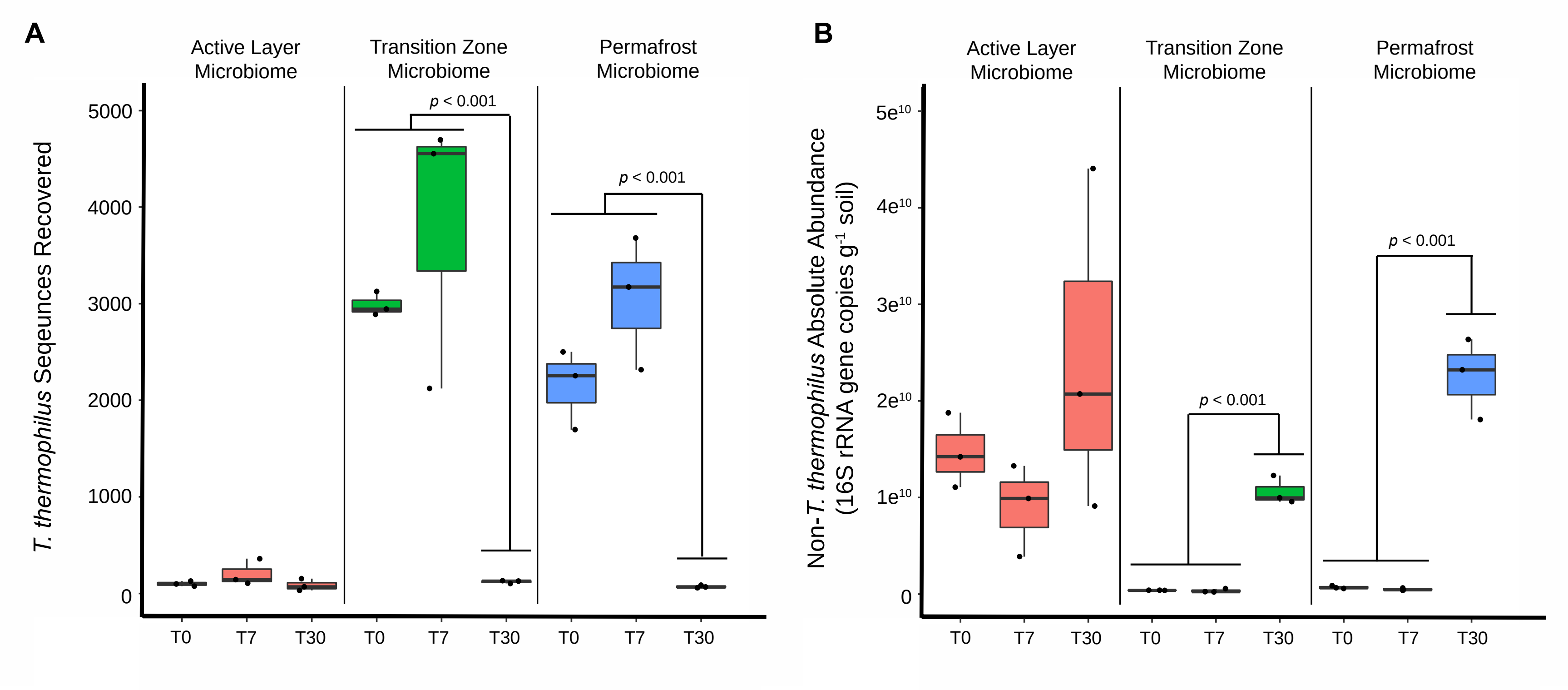

Fig. S2. Boxplot representing (**A**) number of *Thermus thermophilus* internal standard sequences recovered and (**B**) absolute abundance of 16S rRNA gene copies (g^{-1} soil) for non-*T. thermophilus* sequences determined from the recovery of *T. thermophilus* internal standard sequences spiked into each sample prior to genomic DNA extraction. Note the difference in y-axis absolute abundance values between panels. Significant differences were determined between incubation time points within each soil layer microbiome (ANOVA).

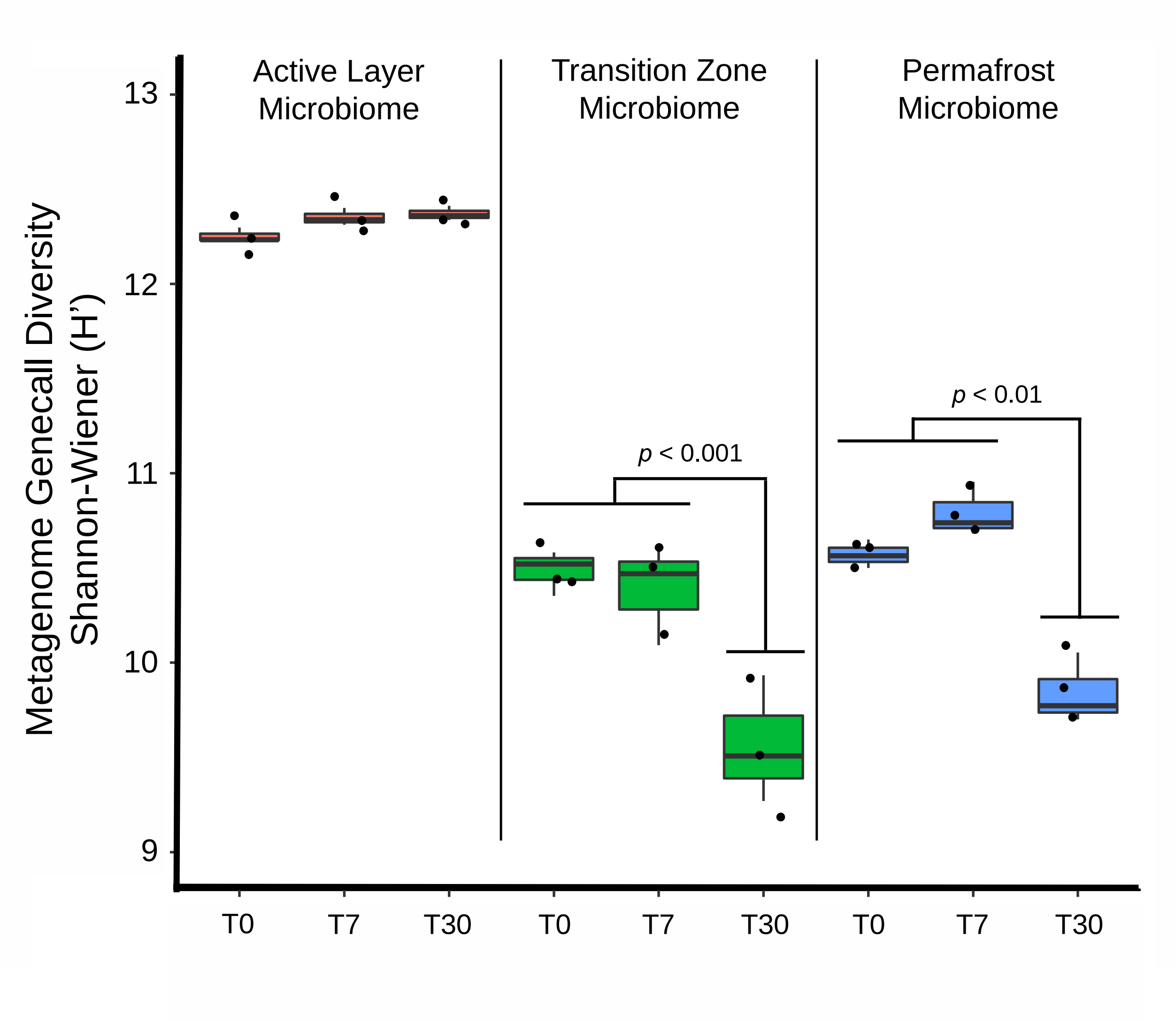

Fig. S3. Boxplot representing the alpha diversity (mean H' ± SD) of functional genes in the metagenome based on the Shannon diversity metric (H'). Significant differences were determined between incubation time points within each soil layer microbiome (ANOVA).

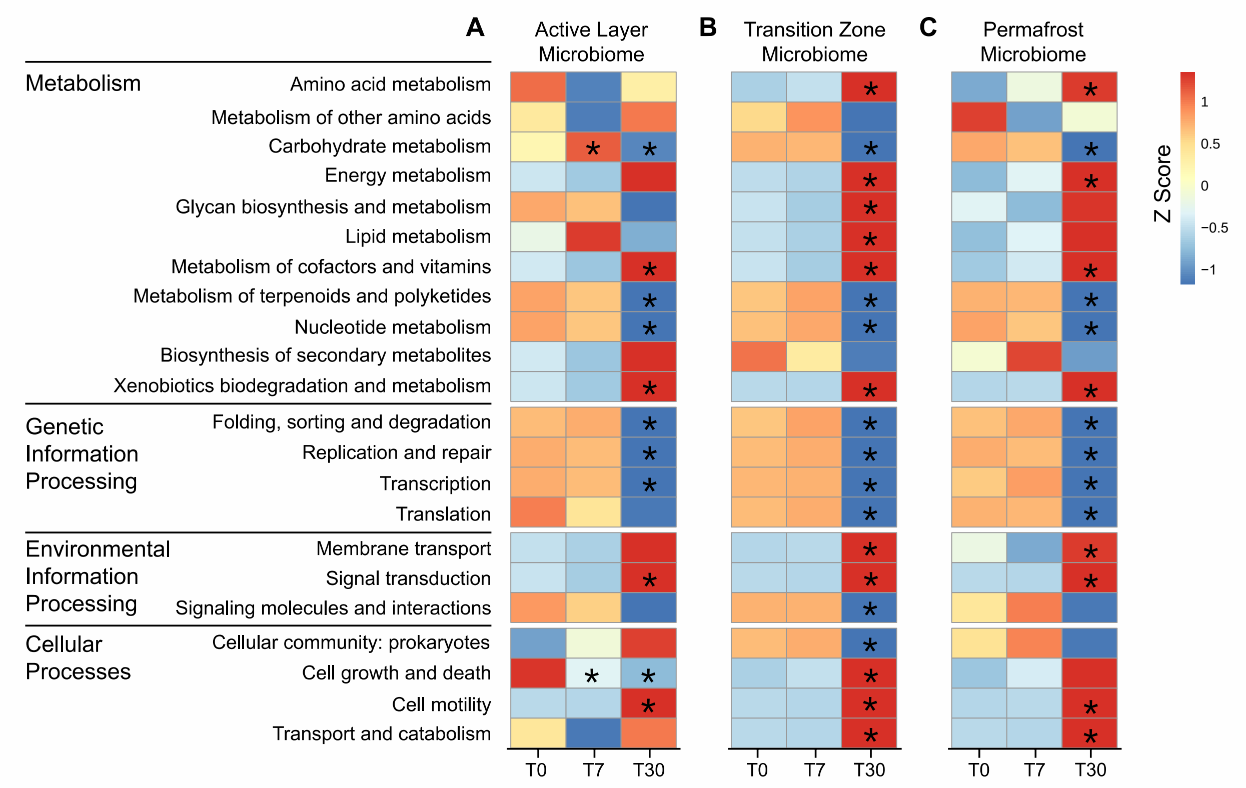

Fig. S4. Heatmap of genomic potential in the (**A**) active-layer microbiome, (**B**) transition-zone microbiome, and (**C**) permafrost microbiome based on Z-score transformations of the mean relative abundance of GPM-normalized metagenome genecalls belonging to KEGG tier III functional pathways for each incubation time point within each soil layer microbiome. An asterisk indicates a significant change ($p < 0.05$) in mean relative abundance of genecalls for each KEGG pathway compared to incubation time point T0 within each soil layer microbiome.
